# Narratives of women presenting with abortion complications in Southwestern Nigeria: A qualitative study

**DOI:** 10.1371/journal.pone.0217616

**Published:** 2019-05-29

**Authors:** Agnes A. Oyeniran, Folasade A. Bello, Babawale Oluborode, Ibraheem Awowole, Olabisi M. Loto, Theresa A. Irinyenikan, Adetokunbo O. Fabamwo, Lanre Olutayo, Bela Ganatra, Philip Guest, Bukola Fawole

**Affiliations:** 1 Department of Health Promotion and Education, College of Medicine, University of Ibadan, Ibadan, Nigeria; 2 Department of Obstetrics & Gynaecology, College of Medicine, University of Ibadan, Ibadan, Nigeria; 3 Department of Obstetrics & Gynaecology, Obafemi Awolowo University, Ile-Ife, Nigeria; 4 Department of Obstetrics & Gynaecology, State Specialist Hospital, Akure, Nigeria; 5 Department of Obstetrics & Gynaecology, Lagos State University College of Medicine, Ikeja, Lagos, Nigeria; 6 Department of Sociology, Faculty of The Social Sciences, University of Ibadan, Ibadan, Nigeria; 7 UNDP-UNFPA-UNICEF-WHO-World Bank Special Programme of Research, Development and Research Training in Human Reproduction (HRP), World Health Organization, Geneva, Switzerland; 8 Institute for Population and Social Research, Salaya, Bangkok, Thailand; Aga Khan University, KENYA

## Abstract

Unsafe abortion continues to impact negatively on women’s health in countries with restrictive abortion laws. It remains one of the leading causes of maternal mortality and morbidity. Paradoxically, modern contraceptive prevalence remains low and the unmet need for contraception continues to mirror unwanted pregnancy rates in many countries within sub-Saharan Africa. This qualitative study assessed women’s knowledge; their expectation and experiences of the methods employed for abortion; and their health care-seeking decisions following a complicated abortion. Women who presented with abortion complications were purposively sampled from seven health facilities in south-west Nigeria. In-depth interviews were conducted by social scientists with the aid of a semi-structured interview guide. Coding schemes were developed and content analysis was performed with WEFTQDA software. Thirty-one women were interviewed. Misoprostol was used by 16 women; 15 women used other methods. About one-fifth of respondents were aged ≤ 20 years; almost one-third were students. Common reasons for terminating a pregnancy were: “too young/still in school/training”; “has enough number of children”; “last baby too young” and “still breastfeeding”. Women had little knowledge about methods used. Friends, nurses or pharmacists were the commonest sources of information. Awareness about use of misoprostol for abortion among women was high. Women used misoprostol to initiate an abortion and were often disappointed if misoprostol did not complete the abortion process. Given its clandestine manner, women were financially exploited by the abortion providers and only presented to hospitals for post-abortion care as a last resort. Women’s narratives of their abortion experience highlight the difficulties and risks women encounter to safeguard and protect their sexual and reproductive health. To reduce unsafe abortion therefore, urgent and synergized efforts are required to promote prompt access to family planning and post-abortion care services.

## Introduction

Unsafe abortion accounts for a significant proportion of maternal morbidity and mortality [1,2]. Between 2010 and 2014, 25% of all pregnancies ended in an induced abortion and on the average, 56 million induced abortions occurred annually [3]. Within the same period, global annual estimate of unsafe abortion was around 25 million, with 97% of these in developing countries. Sub-Saharan African women had the highest risk of dying from an unsafe abortion [2], with the sub-region accounting for an estimated 125,000 deaths [4].

High prevalence of unintended pregnancy, which is the reason for most abortions, has been reported in several studies from Nigeria: 21.0% of women attending antenatal care in Lagos did not want their current pregnancy [5], while 26.6% of women in a community-based study in Lagos and Edo States [6], 29.8% of artisan women in Ibadan [7] and 35.9% of women in rural and urban Ogun State [8] had ever had unwanted pregnancies. In Bankole et al.’s 2006 report [9], which was largely based on data from three surveys (community-based survey, hospital-based survey and 2003 NDHS), 28.0% of all women interviewed had had unwanted pregnancies at some point. It is not all unwanted pregnancies that are aborted, yet these figures suggest that there are a significant number of abortion seekers in these regions. Use of modern contraception would reduce these numbers, but 27.0% of sexually-active women were not on any contraception despite being undesirous of pregnancy [9].

Empirical evidence shows that abortions are quite common and widely practiced [10]. However, according to Nigeria’s bifurcated criminal and penal codes [11], induced abortion is legally permitted only in cases where it is necessary to save the woman’s life. A woman who violates this law is subject to a jail term of up to seven years, while the provider, if convicted is subject to a jail term of up to 14 years. Consequently, they are performed clandestinely or by unskilled providers and are mostly unsafe [12], leading to high maternal morbidity and mortality [13]. In Nigeria, it is estimated that 1.25 million induced abortions occurred in 2012, with wide variations across the country’s geo-political zones and an estimated 212, 000 women were treated for complications of unsafe abortion [12].

Globally, unsafe abortion remains one of the most neglected sexual and reproductive health problems [1]. Complications arising from unsafe abortion especially those considered least safe by WHO (i.e. using traditional invasive methods and provided by persons who are not trained) (2), are numerous and may affect women’s quality of life and well-being. They are sometimes life-threatening, resulting in incomplete abortion, hemorrhage, infection, uterine perforation, damage to the genital tract and internal organs and even death [3]. A facility-based study in South-South Nigeria on the contribution of induced abortion to maternal mortality reported that complications due to induced abortion accounted for 22.8% of the 92 maternal deaths during the study period [13]. The contribution of unsafe abortion to maternal mortality in Nigeria had been reported to be as high as 30–40% [14].

Previous initiatives and studies have targeted the prevention of unwanted pregnancy [15,16], improved access to post-abortion care and strengthening capacity for post-abortion care services [16–21]. Exploring the perspectives of women who experienced abortion has however not received sufficient research attention. Hence, the aim of this study was to document women’s self-narrated experiences of induced abortion, in order to assess their knowledge of this aspect of sexual and reproductive health and understand their pattern of help-seeking behavior and decision making following an abortion complication. The study was part of the World Health Organization’s study of the relationship between the use of misoprostol and the type and severity of abortion symptoms in five countries namely Ghana, Lao PDR, Myanmar, Nigeria and Sri Lanka. This paper reports findings from Nigeria. The other publications from the study evaluated the experience of abortion seekers in Myanmar [22] and perception of service providers in Sri Lanka [23]. Two cross-sectional studies assessed the association between misoprostol and abortion complications [24,25].

## Materials and methods

### Study sites

The Nigerian arm of the study was conducted in nine health facilities (six secondary and three tertiary), in the south-west geo-political zone of Nigeria, where it was estimated that 164,000 induced abortions occurred in 2012 and represents an induced abortion rate of 27 per 1,000 women aged 15–49 years [12]. During the same period in the same geo-political zone, 40% of 59,173 women were treated for abortion complications from induced abortion, rather than miscarriages [12]. The participating health facilities (eight public and one private) were selected based on the availability of a high number of qualified multidisciplinary, full-time medical personnel, a high number of people who utilize the hospitals for sexual and reproductive health issues (especially post-abortion care) and the proximity of the hospitals to the coordinating centre. Five out of the six states within the southwest geopolitical zone of Nigeria were represented in the study.

The qualitative aspect of the study was conducted in seven out of the nine health facilities.

### Study participants, recruitment and sampling

Study participants were women who presented with complications of induced abortion in the seven health facilities. The participants were identified by their health provider as having undergone an induced abortion that required admission to the hospital and the method of the abortion and were then enrolled while on admission in these hospitals, after their treatment was complete and they were stable. Purposive sampling technique was used to recruit participants into the study and the trained interviewer obtained written consent and conducted in-depth interviews (IDIs) with the participants. Although a random selection was not employed due to the characteristics of eligible study participants, recruitment of participants continued till saturation of topics were attained. Only two women among those approached to be interviewed refused to participate in the study.

### Study instrument

An IDI guide (Table 1) was developed by the study team after extensive literature review and consultation with content experts. The guide was semi-structured, facilitating comparability across IDIs and allowing participants to guide the discussion based on their experiences. Four domains of interest, comprising 14 questions were explored: (1) Introductory question (reasons for coming to the hospital and experience); (2) Choice of abortion methods; (3) Care seeking behavior; and (4) Knowledge of methods.

**Table 1:** In-depth interview guide for women who have undergone an induced abortion.

Domains	Questions
A. Introductory questions (warm up)	Reasons for coming to the hospital
	Experience
B. Choice of abortion method	Number of methods
	Type of methods
	Expectation about methods
	Cost and ease of use of methods
	How the last method was used
C. Care-seeking behavior	When was the decision made to seek care
	Where and number of times care was sought
	Visiting hospital part of expectations of use
D. Knowledge of methods	Information available before use
	Information available after use
	Who provided the information
	Information required

### Data collection and management

Research assistants (RAs) were two female postgraduate social scientists from the University of Ibadan and the Lagos State University and two staff members of the University College Hospital (UCH), Ibadan. All four RAs participated in a five-day qualitative research training workshop from October 8–12, 2012 prior to the commencement of data collection in order to develop their research skills and capacity on qualitative methods of data collection. The training was facilitated by an international consultant engaged by the WHO. The training curriculum focused on interviewing techniques, development of coding scheme, coding of transcripts and use of WEFTQDA software. All RAs signed data confidentiality agreements after the training.

One of the two social scientists conducted the face-to-face interviews while the other was the note taker, who recorded participants’ responses on papers and audiotapes during the interviews. Medical officers at the study sites identified eligible women and thereafter informed the research team to move to the study sites for the interviews. Written informed consent was obtained from all participants by the RAs prior to participation. They were provided with information about the purpose of the study and were given ample time to read the consent form before obtaining their written consent. If required, the consent forms were read to them. Consent forms and IDI guides were available in both English and Yoruba (the local language in south-west Nigeria). All IDIs were conducted by RAs during the period of the women’s admission in the hospitals and in specifically designated rooms at all sites to ensure privacy and confidentiality (only the participant and RAs were present). The IDIs, which were conducted in English or Yoruba, were double audio-recorded and each IDI lasted 30 to 45 minutes.

Data were collected from May 2013 to May 2014, until thematic saturation was reached. The IDIs were conducted in the seven hospitals based on the availability of eligible participants during the period of data collection. The audio recordings were transcribed daily by the RAs, and the transcripts and recorded notes were compared for any missing information and updated appropriately. All the IDIs conducted were transcribed in the language of the interview, and those conducted in Yoruba were translated simultaneously into English after the interview. Identifiers were expunged from the transcripts and the de-identified transcripts (in plain text format) were stored on a password-protected computer system.

### Data analysis

The study employed a cross-case content and narrative analysis. The data were analyzed thematically in order to ensure flexibility that assures identification of key themes and sub themes, as well as comparison of similarities and differences in participants’ experiences of induced abortion. Coding schemes were developed independently by two of the authors through a line-by-line coding of a representative sample of the transcripts, selected proportionately through stratification of the number of participants per health facility. Following this process, the similarities and differences in the coding were compared and discussed. A consensus, which included the use of the explanatory thematic framework was thereafter reached and the framework was employed to structure and define the data. Thereafter, the plain text transcripts were imported into qualitative software (WEFTQDA) and categories and subcategories were created in the categories tree using a combined inductive and deductive approach, which used the interview guide and main research questions (deductive) and themes emerging naturally from the data (inductive) to develop the analysis. The transcript texts were coded by marking text passages from the transcripts as related to each of the created categories and all the document sections coded by each category were reviewed side-by-side to allow for comparison of similarities and differences. Narrative texts and illustrative quotes were used to identify patterns in the data and respondent clusters (related themes), and to draw connections between recurrent themes and distinct patterns as they evolved from the richness of the data.

### Ethical and institutional approvals

Approval to conduct the study was obtained from the Institutional Ethics and Research Boards covering all the health institutions where the study was conducted (approval reference numbers inserted in parentheses); University of Ibadan/University College Hospital Ethics Review Committee (UI/UCH/11/0258), Lagos State University Teaching Hospital Health Research and Ethics Committee (LREC/10/06/228), Obafemi Awolowo University Teaching Hospitals Complex Ethics Committee (ERC/2012/12/01) and Oyo State Ethical Review Committee (AD13/479/257). Administrative approvals were also obtained from the participating health facilities. This paper is reported in accordance with the consolidated criteria for reporting qualitative research (COREQ) [26]

## Results

Thirty-one women were interviewed, and all transcripts were included in this analysis. Majority of the participants were aged 21 to 30 years (58.1%), almost half (48.4%) were nulliparous and about one-third (29.1%) were students.

This analysis focuses broadly on the women’s abortion experience, choice of abortion methods, knowledge of methods and health care seeking decision process following an abortion complication. Illustrative quotes were used as relevant to the findings being elaborated upon.

### Reasons for terminating pregnancy

Participants noted four common reasons for terminating their pregnancy. Some of them adduced more than one reason for this decision:

1. Too young/still in school/training/not ready:

Irrespective of their demographic status, participants cited this reason for terminating their pregnancy and some of them further expressed the personal consequences associated with continuing the pregnancy. Specifically, all the student participants were among those who articulated this reason. According to a 20-year-old Artisan and mother of one, she *“wasn’t ready then and there was no money”*.

A 23-year-old student also stated that when she got pregnant, she felt like she didn’t want a baby yet, so she tried to abort the pregnancy. Another student remarked that: “*It is not yet time for me to have a baby…because I am still young and I want to further my education”* [21 years old, Student].

The influence of some participants’ significant others was a factor in the decision to have an abortion. For these women, their partners clearly expressed that they were not ready to have children. Whereas one of these women remarked that her partner expressed this non-readiness prior to getting pregnant: “*when I had my first pregnancy*, *I didn’t tell my husband about it*, *so I went to abort it…I thought he was going to get angry because he already told me that he is not ready for pregnancy”* [22 years old, Nurse], another stated that when she told her partner, he “*accepted it but he said he didn’t want it because he had no money”* [20 years old, Student].

2. Have had the desired number of children:

Some women terminated their pregnancy because either they or their partners did not want more children. Some of these women offered explanation on why they did not want more children: “*my husband said he doesn’t want any more children that we should stop it…I had to do something about it*, *I aborted it”* [36 years old, Artisan]. Another participant perceived that she would be stigmatized, in form of verbal abuse, if people became aware of her pregnancy:

*R*: *I have had the children I wanted and I don’t want any more children…what I was just considering was that people would start asking that what else am I looking for* [37 years old, Trader].

3. Last baby too young and still breastfeeding:

Becoming pregnant while still nursing a child was a reason adduced by some participants for terminating their pregnancy, with one participant associating this with her inability to cope with the care of the children coupled with the effect this would have on her job:

*R*: *I terminated the pregnancy there because I was still nursing a baby and the child was just about 9 months still breast feeding*. *So when I saw that*, *I couldn’t cope with both*, *I didn’t want the child to be affected so I terminated the pregnancy*.

*I*: *When you got pregnant, you were sure you didn’t want it right?*

*R*: *Yes…It wasn’t like I really wanted to abort it*, *I did it because of work*, *I didn’t want it to affect my job* [37 years old, Book keeper].

4. Fear of congenital deformation:

Some participants were on medication when they found out they were pregnant, the fear of bringing forth a deformed child informed their reasons to terminate such pregnancy.

*R*: *…I had preeclampsia [*during previous pregnancy*] so I knew I wasn’t ready for another child yet*. *So when noticed I missed my period*, *I told my husband and he wanted me to keep it*, *but I told him I have used this and that*, *I don’t want a deformed baby* [34 years old, Trader]

Another 34-year-old mother of two said:

*R*: *I had used a number of drugs for boil [*abscess*]; also used Gynaecosid [*drug indicated for the treatment of secondary amenorrhea which produces a bleed*] to prevent pregnancy*. *When I missed my period*, *I told my husband that I did not want a deformed baby*. *I did a pregnancy test*, *which was positive*. *I therefore used misoprostol*.

### Choice of abortion methods

Sixteen (51.6%) and 11 (35.5%) women respectively used misoprostol and D & C to terminate their pregnancy, whereas four women used other methods.

*R*: *So when I had made the mistake and he [*her husband*] said he doesn’t want any more children*, *I had to do something about it*, *I aborted it with D & C*, *I didn’t use drugs* [36 years old, Artisan].

In their attempts to successfully terminate their pregnancy, while some participants used only one method, others employed the use of different methods and/or drugs and were spurred by the *“desperate”* need to get rid of the pregnancy, previous successful termination or recommendations by significant others. Among these participants, some used more than one method to induce abortion. These women procured their abortion through D & C following a failed attempt using drugs. One participant described this: *“it was terminated with D & C but firstly*, *I used drugs*, *it didn’t work*, *so I went for the D & C”* [20 years old, Student]. Another described in detail the “mixed drugs” she used prior to having the D & C:

*I*: *Apart from the D & C, what other methods have you tried?*

*R*: *I used drugs, those ones they mix together…Buscopan, Flagyl, Chloramphenicol and some others*

*I*: *Have you ever used those drugs to induce abortion?*

*R*: *Yes*, *may God forgive me…The first time I used it*, *the blood came and there was no problem*, *but this time it seems different* [26 years old, Trader].

Some other participants used up to two different drugs to achieve their pregnancy termination, including drugs that are not indicated for abortion like postinor 2: “*I took the Postinor last month and I just take* [sic] *Cytotec [*misoprostol*] this month”* [25 years old, Trader]; gynaecosid:*“So when I used the gynaecosid*, *the thing wasn’t coming down and I had a lot of plans before me*, *so I had to seek that friend’s advice and she told me to use misoprostol”* [28 years old, Nurse]; and Alabukun and Andrew liver salt: *“…I know is alabukun and Andrew liver salt and she said I should use it with Regal dry gin*, *but the rest of the drugs I don’t know their names”* [19 years old, Student].

For some of the participants who used various drugs to either prevent pregnancy or induce their abortion, previous “successful” use influenced their perceptions that these drugs are effective. Nevertheless, these perceptions were incorrect and misleading for reasons including the timing of use and the drugs not indicated for the purpose it was used. Hinging on a previous perception of successful use of gynaecosid as an emergency contraceptive following unprotected sexual intercourse, a participant who ended up using misoprostol to terminate her pregnancy remarked that:

*R*: *when my husband and I had intercourse*, *I sensed that I wasn’t in my safe period so I decided to use a preventive measure by taking a drug*. *I took gynaecosid so after a while I found that I have missed my period…* [34 years old, Trader].

### Cost of methods used

The cost of treatment varied and was dependent on the type, number of methods/drugs used, dosage of drugs procured and the source of procurement. Participants who underwent D & C paid between two thousand (about USD 10.00, at the time of the study) and ten thousand Naira (about USD 50.00) while those who used misoprostol paid between eight hundred (about USD 4.00) and eight thousand Naira (about USD 40.00). Other participants who mixed drugs or used drugs like gynaecosid spent between one hundred (about USD 0.50) and two hundred and seventy Naira (about USD 1.35) for treatment. Women often paid for treatment by themselves, while a few of them stated that their husband or boyfriend paid.

### Knowledge and use of abortion methods

#### Women’s sources of information and procurement

A variety of people assisted participants to terminate their pregnancy, and either served as source of information or procurement of the abortion methods. Sources of initial information were friends, internet, colleagues and patent medicine vendors while sources of procurement included the friends, patent medicine vendors, nurses, doctors and pharmacists. It is however unclear whether the aforementioned medical personnel were qualified or not.

*R*: *I searched on internet for drugs to abort*, *Google…I typed abortion drugs*, *a lot of drugs*, *a lot* [21 years old, Student].

*R*: *Yes*, *I went to a chemist*. *I had already bought drugs*, *used them and started bleeding before I came here* [20 years old, Artisan].

*R: I sought advice because I actually thought I would find a drug to use when I discovered I was pregnant so I was told that drugs can’t abort pregnancies of such weeks…9 weeks 3 days. So I was told that D & C would be preferable since I couldn’t cope with it*.

*I*: *Who gave that information?*

*R*: *We work together*, *she’s like a senior colleague to me* [37 years old, Book keeper].

When source of information or procurement was health workers, little or no information was provided on the procedure or mode of action. This sentiment was particularly echoed by participants who procured their abortion through D & C. For example, when asked if the nurse told her anything before she started the D & C, a women answered in the negative: “*No*, *she didn’t*, *she just told me to lie down and later*, *she told me that the thing has started developing and that was just all”* [25 years old, Trader]. In addition, these women reported that the procedure was performed in private clinics, homes or chemists, supposedly by either a nurse or a doctor. Some of these women further related their uncertainty about the professional identity of their abortion providers. For example, three participants remarked that they identified the provider as a doctor because *“he worked as a doctor”* [20 years old, Student], the person who recommended the provider *“said the person is a doctor”* [24 years old, Artisan] and “*he has the instruments…people refer to him as doctor”* [20 years old, Student].

#### Information available before and during use and how methods were used

Only a few of the participants knew the duration of the pregnancy when they terminated it; a very important piece of information needed to ascertain the appropriate abortion method. For women who knew, the gestational age was between one and three months. Generally, women had little knowledge about methods used; many of them did not know the names of the drugs or procedure. Participants that were aware of misoprostol for abortion had little other information about its use. Some who used misoprostol did not know the name of the drug but described it with expressions like: “*there were 3 little white drug with edges*” [20 years old, Artisan] and *“it was white*, *it has a star shape and it was little”* [21 years old, Student]. The few who knew the drug names and information about the uses was because of their type of occupation as a nurse or patent medicine vendor: “*gynaecosid is used in amenorrhea when you are not seeing your period and it is not meant for pregnant women but can also be used in pregnancy to induce abortion; it’s (misoprostol) for 2 purposes*, *one patients having miscarried can use it and also for abortion”* [28 years old, Nurse]. Relatedly, women who had D & C were unable to vividly describe the procedure save for being told to lie down, given an injection and insertion of instrument into the vagina. Only two of these women were able to provide a little more information which included being showed blood and use of “*a metallic object*, *passed through vagina to bring out a tiny stuff* or *substances”*, representing the evacuated product of conception.

Except for the women who already had information about the drug they require, it was rather common for women to “*just explain*” to the providers that they needed to terminate their pregnancy without asking “*particularly” for a method or drug*. Despite a previous abortion in which a participant had used misoprostol, the participant was unable to mention and describe the drug used to terminate the recent pregnancy:

*I*: *What drug did you use then?*

*R*: *I can’t say precisely because the pregnancy was about 3 weeks plus then*, *so I took “apiol and steel” [*a toxic abortifacient*] it didn’t work*, *then later I took misoprostol or something prostol*, *I can’t remember vividly*.

*I:* *Alright, let’s come back to this current experience…*

*R:* *…I took a drug, I don’t know the name of the drug precisely…*

*I:* *…could it have been misoprostol?*

*R*: *No*, *because the doctor asked me if I had ever taken drugs*, *I said yes*. *I only inserted it* [20 years old, Student].

Women who used drugs to terminate their pregnancy used varying dosages, depending on a number of factors including their source of information/procurement and their feelings about the drug. They were often required to use multiple routes of drug administration. Oral administration of these drugs was easy for the women. Some of them were however afraid to administer misoprostol vaginally and they felt that it was inconvenient to use. When women had conflicts between the administration instructions and how they felt about the administration, they often opted for oral. This sentiment was described by a 23-year-old student who obtained six misoprostol tablets from a chemist and was instructed to place two tablets each under the tongue at 3 hours interval and insert the last two tablets vaginally. She was however scared of the vaginal administration, and consequently used all tablets sublingually.

Another participant—a 21-year-old student—who also used misoprostol but obtained her information through the internet, detailed the information she obtained which included how to use it, including dosage and a pictorial description. Despite the information she obtained on the dosage through the internet, she reported that she used the drug differently from the information she found on the internet. She stated that she took the drug twice, at an interval of about 8 days. She placed the drug under the tongue on both occasions, and once the drug had dissolved, swallowed the remaining fragment with lime and gin. She admitted that she used the drug differently because she didn’t have adequate knowledge about the drug.

Similarly, other participants offered differing explanation on how they administered the drugs used. While one of our participants who used misoprostol *“used all the three tablets once*, *with water”* [20 years old, Artisan], another used “*4 tablets per vagina”* and *“had already used gynaecosid*, *two tablets once” a day prior* [28 years old, Nurse]. A 23-year-old patent medicine vendor, who used gynaecosid remarked that she followed the prescription written on it and explained that she “*used one tablet with one sachet of Seaman’s schnapps*. *According to the prescription*, *one tablet should be taken then 6 days later*, *if there are no effects the second tablet should be used”*. A participant who used a combination of medications described how she used the drugs, in accordance with the provider’s instructions:

*R*: *The alabukun and the Andrew liver salt*, *she said I should use regal dry gin to swallow it*, *but for the rest of the drugs*, *she told me to insert them into my vagina around 10pm…2 tablets once*, *before I sleep*. *For the other drugs*, *she said*, *I should use it 6am when I wake up* [19 years old, Student].

A participant who remarked that misoprostol was not convenient to use said that she was instructed to use candle to insert it into her vagina secretly and without help: “S*he said I should do it myself*, *that I should not let anybody know; I should put the drug inside my private part*, *then use the candle to push it in”* [19 years old, Student]. While comparing the ease of using the medical and surgical abortion methods, a woman who used both misoprostol and D & C stated that *“none of them was easy to use but the D & C was very painful”* [20 years old, Student].

#### Information available after use

Some of the participants obtained information regarding the abortion methods they used after they had experienced and presented themselves in the hospital for complications. As part of history taking, participants stated that they were asked to mention the abortion procedure and medications they had used; which a number of them described, for the reason that they did not know the name of the drug or procedure. Whereas some of the information women received on the drugs and procedure were direct, others were obtained indirectly by listening on the conversation of the health workers.

*R*: *Yes*, *when I was asked*, *I explained that it was white and small so they called it Cytotec*. *I heard that from the nurses’ discussion*. *They said the drug is so effective that even if they are to prescribe it to their patients*, *they would prescribe it for pregnant women* [23 years old, Student].

Generally, information obtained by participants from the hospital were the names of the drugs and procedure, indications, effectiveness and use. Women who used misoprostol were told that it is for pregnant women in labour, and that the pill would *“push out babies”*. The information provided to women on the use of misoprostol centered around childbirth, albeit with varied dosages of a quarter to half tablet. None of these participants stated that they received information on the appropriate misoprostol dosage for abortion.

#### Women’s expectations and experience with abortion method used

Overall, women had much confidence in the efficacy of the abortion methods and did not expect a method failure or complications. A 21-year-old student reinforced this confidence and remarked that she was given “*90% assurance that it would work and that if it doesn’t work that [she] should use it again”*. Women’s expectations following the termination of their pregnancy were formed by the details on the mechanism of action provided by their information sources. Some of the women observed the exact signs that were described by their abortion providers as evidence of successful pregnancy termination; one of this is that *“if it had worked”*, they will see their menstruation and they will bleed. On the other hand, the signs some women experienced following the abortion were in contrast to the information received or their thought. Some of them related their experience thus:

*R*: *When I used it* (gynaecosid), *I felt that even if I will have to come to the hospital*, *the pregnancy would have been terminated or I will have miscarriage or bleeding*. *That was what I thought but when everything was not forthcoming*, *I had to involve my friends* [28 years old, Nurse].

*R*: *My expectation* (misoprostol) *was that I will have abdominal cramp*, *the thing would fall then there will be bleeding but when I used it*, *I had a little bit cramp and I saw some clot and that was all* [28 years old, Nurse].

Generally following the abortion, women experienced pain, fever and bleeding, which ranged from mild to severe. They likened the pains to postpartum pains and described it as *“a little abdominal discomfort”*, unpleasurable, *“really unbearable”* which was felt all over the body, and *“severe”* to the extent that it was impossible to sleep. Women shared related experiences irrespective of the abortion method used to end their pregnancy, but women who underwent D & C described greater pain intensity. For instance, a participant who used misoprostol and had to be transfused with blood described thus: *“It caused me headache*, *pain*, *bleeding (I lost blood) and I fell sick”* [23 years old, Student]. Comparably, a 26-year-old trader, who had D & C stated that she experienced pains all over her body, *“couldn’t bend down”* and three days after the procedure she began to experience severe bleeding.

Following their experience, women, especially those who aborted their pregnancy through D & C, remarked that they would not wish to use the method again because of pains they experienced. Although a few women noted their preference for misoprostol over D & C, most of them were generally disappointed with their experience since the methods did not complete the abortion process as expected. They consequently expressed hesitation about future pregnancy termination and recommending the methods they used to other women. One of the women who indicated this hesitation further noted that since she *“didn’t make the mistake publicly*, *only [her] husband knows about it*, *so no one would ask”* [36 years old, Artisan]. While also expressing reservations about recommending the mixed drugs she used to a friend, a participant linked such action to possible loss of live and consequent arrest by the law enforcement agency:

*R*: “*Ah*! *You know I will be the one they will arrest*, *let’s assume they also do not have money like my parents*, *the person will just die and I will be arrested”* [26 years old, Trader].

One of the women [37 years old, Book keeper] who aborted her pregnancy through D & C stated that for her to recommend the method to a friend, she would “*make her understand that it is painful*, *so she should think about it properly*, *if she wants to do it*, *she shouldn’t go to quacks”*.

### Care seeking behavior

Visiting hospital for post-abortion care was not part of the expectations of use because they “*never even thought there will be consequences”*. Majority of the respondents did not envisage that they would have to visit the hospital for evacuation or other post-abortion care after the D & C or after the use of misoprostol as prescribed. One of the participants who obtained the abortion drug from her husband—who is a medical practitioner—however remarked that she knew she might end up in the hospital for evacuation. Below are some responses why visiting hospital was not part of their expectation.

*R*: *I never thought I will come to (the hospital)*, *with hopes that I will be fine*, *because I sought advice and I was told that that’s her specialization so I never thought so* [37 years old, Book keeper].

*R*: *No*, *even when I was having the bleeding*, *I went to her and she said it would stop that she will give me some drugs*, *vitamin K that it would stop the bleeding*, *she said I should come and buy it in the evening*, *that she doesn’t have it in her shop*, *so before evening*, *I fainted and they rushed me to the hospital* [19 years old, Student].

Since women’s experiences with the abortions were not at par with their expectations, they ended up going to the hospital to seek care. The decision to seek care at the hospital was usually after experiencing complications (abdominal pains, fever, dizziness or continuous bleeding), or indications that the abortion was incomplete. This decision making was commonly a last resort after every other effort to manage it had failed.

*R*: *Although*, *I felt pains that day but I was hoping it was because I just had it*, *the second day*, *I use drugs*, *no changes…the second day after I had it*, *I saw blood and water*, *it was this blood and water that continued to come*, *it didn’t stop*, *later it became black…so on the third day*, *when I saw no improvement I had to come back here* [37 years old, Trader].

*I*: *So when did you decide to come to the hospital?*

*R*: *It was about 3 days at night and I still didn’t get myself*, *we went to a nurses’ home then injected me but instead of getting better*, *it became worse so my mum brought me* [26 years old, Trader].

*R*: *I didn’t have any intention of visiting the hospital but when I began to bleed*, *the blood came gradually*. *About a few weeks later*, *I began to have severe stomach ache*, *my parents thought it was malaria so my younger sister went to get a drug for me at the chemist*, *a white drug sort of*, *that was what I used and I was relieved*. *I couldn’t even check the name because of my condition*. *So when I used it*, *I felt ok for about 2 weeks but on Saturday the pains began again and I was bleeding that is why my grandma brought me here* [23 years old, Chemist].

## Discussion

This study was conducted to explore women’s experiences, knowledge of abortion methods, health care-seeking decisions and needs following an abortion complication in south-western Nigeria. All but one of the women procured an abortion because the pregnancies were unplanned. This pattern cuts across all the women irrespective of their age, occupation, level of education, social and economic status. Given the reasons adduced by participants for pregnancy termination, this study affirms previous reports that unwanted pregnancy is the underlying cause of induced abortion [6,9]. This phenomenon reflects the problem women face in regulating their childbearing; either to delay starting a family, postpone next birth or to cease childbearing. The reasons women gave for not wanting their pregnancy is consistent with findings which noted that these vary with women’s life circumstances [6,8,9]. This finding calls attention to the unmet need for family planning. The use of modern contraception evidently lowers the incidence and prevalence of induced abortion and meeting the unmet need for family planning is an effective action to reduce unintended pregnancy and induced abortion [27].

Expectedly, participants procured abortion in clandestine manner using misoprostol, other drugs or through D & C and sometimes a combination of both, which was provided or recommended by nurses, doctors, patent medicine vendors and friends in private settings. This could be attributed to Nigeria’s restrictive abortion laws. It is worrisome however that some participants used various drugs and unconventional preparations like gynaecosid (oestrogen), postinor-2 (levonorgestrel; Plan B) and mixed drugs [(buscopan (hyoscine butylbromide), flagyl (metronidazole), chloramphenicol, Alabukun (acetylsalicylic acid and caffeine), Andrews liver salt (magnesium sulphate powder), Seaman’s schnapps (alcoholic drink) and dry gin] to terminate their pregnancies. Similar findings were reported among those who sought abortion with medication in South-East Nigeria [28]. For instance, postinor-2 is an emergency contraceptive pill which may be taken immediately after or up to 120 hours after unprotected sexual intercourse [29]. In this study, participants used postinor to unsuccessfully attempt abortion after they had missed their period, with a gestational age of up to two months, rather than as a contraceptive.

The use of various unusual abortion methods might be an indication that some of the abortionists were untrained or unqualified, and it also underscores why the women might have experienced complications. Some of the participants in this study remarked that their pregnancy was terminated by ‘doctors’ and ‘nurses’. With a widespread assumption that people wearing white coat or nurse gown design must be doctors or nurses, there is no way to substantiate that these medical personnel were qualified and have the requisite training. Experiential knowledge of Nigeria indicates that clinics are sometimes manned by auxiliary nurses or technicians. Also, patent medicine stores (“chemists”) are not usually covered by pharmacists. As with clandestine abortion, abortions that were performed by untrained persons may have been performed without proper medical guidance and follow-up and use of appropriate aseptic techniques. This finding agrees with other studies in Nigeria [13] and Ghana [30].

The varied cost of treatment in this study provides an important insight into the manner in which women who undergo abortion are exploited by the providers. This finding on financial exploitation of women by abortion providers is in parallel with studies conducted in Sri Lanka [31] and India [32]. Specifically in the Nigerian context, since the procedure itself is legally restricted and there is no regulatory or institutional oversight, women seeking abortion on request are in a vulnerable position. Their desperation to have the procedure done by all means necessary—with no legal alternatives—while maintaining secrecy is easily preyed on by the providers who in turn profit from this as they would a lucrative business.

Whereas the appropriateness of methods of abortion differ by pregnancy duration [27,33], participants’ choice of abortion methods was made without regards to the duration of their pregnancy. In addition, varying dosages of misoprostol was used by the women to abort their pregnancy irrespective of the duration. Given that they had limited knowledge about the drug and the influence of gestational age on dosage, they depended on their information sources and their personal reasoning. Often, they ingested an under-dose of the drug or used it inappropriately, which apparently marred the effectiveness of the drug, resulting in incomplete abortion and hemorrhage. Similar incorrect dosage was also found in a concurrent study carried out in Myanmar (22). The recommended drugs for induced abortion are mifepristone and misoprostol, which are used in combination or misoprostol used as a stand-alone; though with lower effectiveness [27,33]. Although the incorrect use of misoprostol may not be as unsafe as invasive and traditional methods of abortion, knowledge and correct use of these drugs is important [33], as evident by the complications experienced by some of the women in this study and their need for post-abortion care. Mifepristone was not available in Nigeria, as at the time of the study.

Women’s expectations were not at par with what they experienced following the termination of their pregnancy. As documented in a previous study, women usually expect favourable outcomes but when abortion is unsafe, complications develop which consequently lead to presentation in the hospital for care [13]. This was clearly demonstrated in this study, which left many of them disappointed. On presenting to the hospital, women’s complaints were often vaginal bleeding, fever, abdominal pains and dizziness. This is similar to previous studies conducted in health facilities in the South-South geo-political zone of Nigeria [13,30]. However, in getting help for these sequelae of abortion, many of the women in this study either stayed back at home, went back to the original abortion providers or procured medicines for the presenting indication without taking into account the underlying cause. They eventually presented in the hospital when their clinical situation deteriorated. Hence, visiting the hospital was obviously not part of their expectations. This reluctance to present in the hospital for care is indicative of the secrecy associated with induced abortion, given its illegality in the country and the fear of stigma, punishment and moral reproach [27,34].

Reducing unsafe abortion is a necessary strategy to reduce maternal mortality and achieve the Sustainable Development Goals (SDGs) 3 and 5. The SDG 3 includes targets to reduce maternal mortality ratio to less than 70 per 100,000 live births by 2030; ensure universal access to sexual and reproductive healthcare services; and achieve universal health coverage including access to safe, effective, quality and affordable medicines for all [35]. In like manner, one of the SDG 5 targets is to eliminate all forms of violence against all women and girls in the public and private spheres including sexual and other types of exploitation [35]. Having fallen short of achieving the Millenium Developmental Goal (MDG) 5 targets of reducing maternal mortality and achieving universal access to reproductive health [15], the SDG era provides Nigeria with the unique opportunity to leverage on the experiences of the MDGs implementation and vigorously pursue these SDGs for better outcomes and desirable impacts.

Study limitations include concerns about the accuracy and reliability of information given, based on recall, and because of the sensitive nature of abortions procured illegally. Recall bias will not likely be a significant factor, though, as the events being inquired about are recent. The interviews were carried out after treatment, so it is hoped that the particiapants would have overcome their fears of being arrested or stigmatised, by this time. The findings of this study relate only to a limited number of hospitals and therefore the interpretations must be treated with caution.

## Conclusions

The abortion experiences of women highlight the burden women face to regulate and maintain their fertility and sexual and reproductive health and thus underscores the importance of collective responsibility towards ensuring a reduction in unsafe abortion. In lieu of this, reduction of unsafe abortion as a strategy for improving maternal health would require a robust and comprehensive effort. These strategies would include reducing the recourse to abortion through (1) improving women’s reproductive health knowledge through education; (2) enhancing women-centered uptake of family planning based on their choice of methods; (3) engagement and involvement of women’s significant others in abortion prevention efforts [27,36–38]. Strengthening of post abortion care services to ensure access for all women and quality care is a vital component among measures required to reduce the impact of unsafe abortion on women’s health. Nigerian abortion law reform has been stiffly opposed in the past. Any attempt at reform must seek to advocate to reduce the incidence of unsafe abortion and therefore promote safe motherhood [39].
